# Cofilin Signaling in the CNS Physiology and Neurodegeneration

**DOI:** 10.3390/ijms221910727

**Published:** 2021-10-03

**Authors:** Jannatun Nayem Namme, Asim Kumar Bepari, Hirohide Takebayashi

**Affiliations:** 1 Department of Pharmaceutical Sciences, North South University, Dhaka 1229, Bangladesh; jannatun.namme@northsouth.edu; 2 Division of Neurobiology and Anatomy, Graduate School of Medical and Dental Sciences, Niigata University, Niigata 951-8510, Japan

**Keywords:** cofilin, cofilin-1, cytoskeleton, neurodegeneration, actin, neuron, Alzheimer’s disease, schizophrenia, LIMK1, SSH1

## Abstract

All eukaryotic cells are composed of the cytoskeleton, which plays crucial roles in coordinating diverse cellular functions such as cell division, morphology, migration, macromolecular stabilization, and protein trafficking. The cytoskeleton consists of microtubules, intermediate filaments, and actin filaments. Cofilin, an actin-depolymerizing protein, is indispensable for regulating actin dynamics in the central nervous system (CNS) development and function. Cofilin activities are spatiotemporally orchestrated by numerous extra- and intra-cellular factors. Phosphorylation at Ser-3 by kinases attenuate cofilin’s actin-binding activity. In contrast, dephosphorylation at Ser-3 enhances cofilin-induced actin depolymerization. Cofilin functions are also modulated by various binding partners or reactive oxygen species. Although the mechanism of cofilin-mediated actin dynamics has been known for decades, recent research works are unveiling the profound impacts of cofilin dysregulation in neurodegenerative pathophysiology. For instance, oxidative stress-induced increase in cofilin dephosphorylation is linked to the accumulation of tau tangles and amyloid-beta plaques in Alzheimer’s disease. In Parkinson’s disease, cofilin activation by silencing its upstream kinases increases α-synuclein-fibril entry into the cell. This review describes the molecular mechanism of cofilin-mediated actin dynamics and provides an overview of cofilin’s importance in CNS physiology and pathophysiology.

## 1. Introduction

Neurons contain a cytoskeleton consisting of microtubules, neurofilaments, and actin filaments. Microtubules are composed of tubulin proteins and other polypeptides and provide the essential organization of organelles. Neurofilaments, which are class IV intermediate filaments, offer structural support to axons and influence nerve conduction velocity. Actin filaments (F-actin) are composed of globular actin monomers (G-actin). Dynamic transition can occur between G-actin and F-actin, and the polymerization-depolymerization events are spatiotemporally regulated in response to numerous extracellular and intracellular stimuli. Actin is an ATPase, and both G-actin and F-actin can bind to ATP. When ATP-bound G-actin monomers assemble into a polymer, ATP hydrolyzes rapidly to generate ADP-Pi actin, slowly releasing the inorganic phosphate producing ADP-actin subunits. Consequently, actin filaments exhibit considerable asymmetry with a plus end (barbed end or growing end) and a minus end (pointed end or shrinking end) dominated by ATP- and ADP-actins, respectively. ATP-G-actin monomers are preferentially polymerized to the barbed end whereas, ADP-actin subunits are depolymerized from the pointed end. The actin dynamics is precisely controlled by various actin-binding proteins (ABPs) such as actin-related protein 2/3 complex (Arp2/3), cortactin, formin, profilin, and actin-depolymerizing factor (ADF)/cofilin (reviewed in [1]).

Three ADF/cofilin family members are expressed in mammals: ADF, cofilin-1, and cofilin-2 (reviewed in [2,3]). The first member ADF (also known as destrin), encoded by the gene *DSTN* in humans, was initially identified in the chick brain [4]. Cofilin was discovered as an actin-interacting protein in the porcine brain [5,6]. Later, Ono et al. identified two mammalian variants of cofilin, non-muscle type (also known as cofilin-1 and n-cofilin) and muscle type (also known as cofilin-2 and m-cofilin) [7]. In humans, cofilin-1 and cofilin-2 are encoded by the genes *CFL1* and *CFL2*, respectively. Different isoforms of ADF/cofilin have qualitatively similar but quantitatively different effects on actin dynamics [8]. To be noted, both ADF and cofilin show cooperative binding with actin filaments [9,10]. Interestingly, cofilin-1 comprises almost 90% of the total ADF/cofilin family in CNS [11]. For simplicity, we will use the term ‘cofilin’ to mention cofilin-1 hereafter.

**Figure 1:**
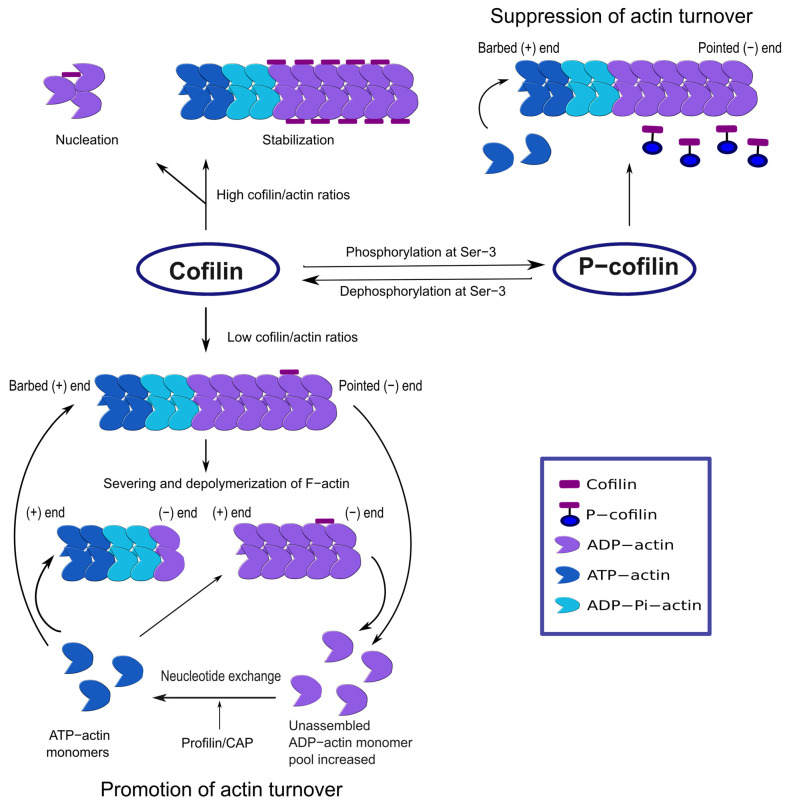
Actin dynamics regulation by cofilin. At low cofilin/actin ratios, cofilin severs F-actin and increases the ADP-actin monomer dissociation rate. Actin polymerization is favored in the presence of nucleotide exchange regulators (e.g., profilin, CAP). At high cofilin/actin ratios, cofilin stabilizes F-actin where all the subunits have undergone cofilin-induced rotation. Cofilin can also induce nucleation. Inactive p-cofilin does not significantly bind to F-actin, and actin severing or depolymerization is low.

Cofilin can bind to both G-actin and F-actin, exhibiting stronger affinities for the ADP-bound actins than the ATP- or ADP-Pi-bound forms [12]. Cofilin binding to F-actin induces actin subunit rotation, enhances Pi release along the filament, and promotes filament severing in a concentration-dependent manner [8,13,14] (Figure 1). Severing is rapid at a low cofilin/actin ratio and suppressed at a high cofilin/actin ratio. Interestingly, only a few cofilin molecules can induce actin filament fragmentation, predominantly at the pointed end of the cofilin domain [15,16]. Severing generates newer ends of the filament where cofilin may accelerate the disassembly of ADP-actins from the pointed end [16]. On the contrary, higher cofilin concentrations can favor actin polymerization through nucleation [13]. Thus, cofilin is capable of controlling actin dynamics through both polymerization and depolymerization.

Conceivably, regulation of cofilin activity is immensely complex where diverse stimuli in the cell microenvironment orchestrate the cytoskeleton dynamics in physiological and pathophysiological conditions [17,18]. For instance, two guidance cues, nerve growth factor (NGF) and netrin-1, were found to activate cofilin, increase free actin barbed ends, and promote growth cone protrusion [19]. Meyer et al. (2005) showed that insulin-like growth factor I (IGF-I) enhances neuroblastoma cell motility through activation of cofilin and its upstream regulators [20]. Tilve and colleagues (2015) observed an extracellular synuclein-induced cofilin inactivation and dysregulation of neuronal actin dynamics [21]. At the molecular level, cofilin activity is modulated by phosphorylation-dephosphorylation, binding to other regulatory proteins, and redox modifications. We have discussed essential cofilin regulators in the next section. For further details, interested readers are referred to many outstanding reviews describing the roles and regulations of cofilin in actin dynamics [22,23,24,25,26,27].

## 2. Signaling Mechanisms for Cofilin Activation and Inactivation

**Figure 2:**
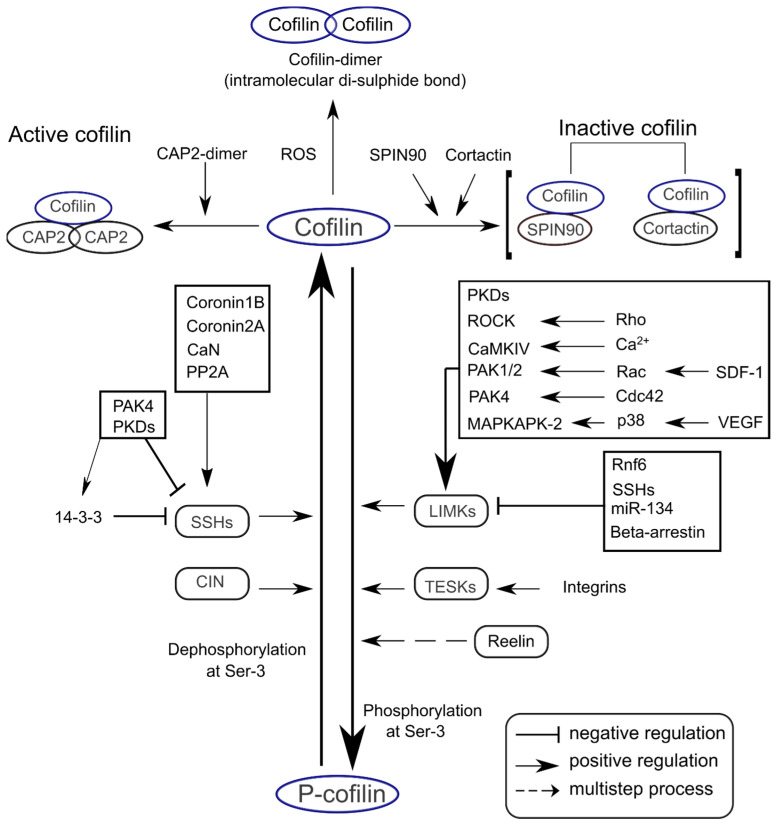
Signaling mechanisms regulating cofilin activity in the CNS. Cofilin can be dephosphorylated at the Ser-3 residue by SSHs and CIN. Cofilin Ser-3 phosphorylation is mediated by LIMKs and TESKs. These phosphatases and kinases can be activated or inhibited by diverse upstream regulators. Cofilin activity is enhanced through binding with CAP2 dimers (upper left), while binding with SPIN90 or cortactin decreases cofilin activity (upper right).

The Ser-3 residue in cofilin is a conserved phosphorylation site [28,29]. Cofilin is activated via dephosphorylation at Ser-3 by slingshot family proteins (SSHs; SSH1, SSH2, and SSH3) through the Ca^2+^/calmodulin-dependent calcineurin activation pathway [30,31]. On the other hand, cofilin is deactivated via phosphorylation at Ser-3 by LIM domain kinases (LIMKs) and dual-specificity testis-specific protein kinases (TESKs) [32,33]. Major proteins modulating cofilin activity are listed in Table 1, and critical signaling mechanisms are summarized in Figure 2.

Though all three mammalian SSHs have cofilin phosphatase activity, SSH1 is the most effective in activating cofilin [30]. The N-terminal domain of SSH1 interacts with cofilin, where the Cys-393 residue of SSH1 is critical in removing the phosphate group from cofilin Ser-3 [51,52]. SSH1 can also dephosphorylate LIMK1 and attenuate LIMK1′s enzymatic activity towards cofilin [53]. Singla et al. (2019) showed that growth factor induction in macrophages can activate cofilin and regulate actin dynamics through an SSH1-dependent pathway [54]. In addition, SSH1 has independent F-actin-stabilizing and bundling activities and promotes the disassembly of F-actin [34].

Coronins are F-actin-binding proteins, which also interact with microtubules and modulate cell motility and actin dynamics. SSH1 can dephosphorylate Coronins. Inversely, coronins may dephosphorylate SSH1 and induce cofilin activation at the leading edge [35,36]. Coronin-1B directs SSH1 towards lamellipodia and thereby regulates the activity of cofilin via dephosphorylation [35]. Coronin-2A binds and colocalizes with SSH1 at focal adhesions [36]. Depletion of coronin-1B inhibits SSH1-induced lamellipodial dynamics and cofilin activation, while coronin-2A depletion increases the p-cofilin level and diminishes cell migration and focal adhesion. Coronin-2A depletion can be rescued by expressing active cofilin in cell-free assays and cultured cells [36].

LIM-kinases are actin-binding proteins, which phosphorylate cofilin specifically at Ser-3, attenuating the actin-binding, severing, and depolymerizing activities of cofilin [32,33], reviewed in [24]. LIMK1 inactivates cofilin under control of ras-related C3 botulinum toxin substrate 1 (Rac1), while LIMK2 phosphorylates cofilin in response to Rho and Cell Division Cycle 42 (Cdc42) rather than Rac1 [42,55]. Downregulation of LIMK1 suppresses the lamellipodium formation induced by Rac1 or insulin. Therefore, both LIMK1 and LIMK2 phosphorylate cofilin through the Rac1/PAK and Rho/Cdc42 pathways, respectively.

TESKs are structurally related to LIM-kinases with a kinase domain and a unique C-terminal proline-rich domain. TESK1 can phosphorylate cofilin at Ser-3 in vitro and in vivo to affect actin organization [44]. When active cofilin was expressed in HeLa cells, rhodamine-phalloidin (a conjugate dye used to stabilize actin filaments in vitro) staining was markedly decreased by cofilin-mediated actin depolymerization, and this phenomenon was reversed by co-expression of TESK1 with cofilin [56]. Using TESK1 knockout mice, Wang et al. (2018) showed that TESK1 kinase activity is critical in cofilin-induced actin depolymerization [57]. TESK2 was also found to mediate cofilin phosphorylation and the formation of actin stress fibers in cultured cells [45].

CAPs are multi-domain actin-binding proteins having the capability of actin dynamics regulation at multiple levels [58]. CAPs and cofilin synergize to enhance depolymerization of F-actin at the pointed end [50]. CAPs compete with cofilin to bind with G-actin and promote its nucleotide exchange. Though two homologs of CAPs, CAP1 and CAP2, have been described in mammals, in humans, CAP2 plays a crucial role in neuronal cells, most notably in spine morphology [59]. CAP2 dimers/oligomers promote depolymerization of cofilin-saturated fragments of F-actin [50,58]. Alterations in dendritic architecture and spine morphology have been reported in CAP2 knockout neurons [60]. A recent study reported CAP2 as a postsynaptic protein relevant to regulating synaptic transmission and plasticity by shaping dendritic and spine morphology, which are all interconnected to actin depolymerization through cofilin activity [60].

In addition to phosphorylation-dephosphorylation, cofilin activity is also altered by several other mechanisms, including redox regulation. ROS can directly modulate cofilin in two different ways. First, direct oxidation of cofilin at Cys-139/147 to sulfenic acid in response to hydrogen peroxide (H_2_O_2_) impairs cofilin binding to actin [61]. Second, under mild oxidative stress, ROS induces cofilin activation and an intermolecular disulfide bond between Cys-147 and Cys-39, forming cofilin-actin oligomers [62,63] and may lead to rod formation under an oxidative environment. Nonetheless, ROS can modulate cofilin by oxidation and inhibiting its actin-severing action.

## 3. Cofilin Functions in the CNS Development

### 3.1. Neural Tube Morphogenesis

Neurulation in human embryos proceeds in two phases, primary and secondary. The neural tube, the embryonic precursor of the CNS, is developed from the neural plate (a section of the ectoderm) via primary neurulation [64] involving four overlapping stages: neural induction, shaping, bending of the neural plate, and neural tube closure [65]. Neural crest cells are generated from the neural tube during neurulation and take long migration routes before settling and differentiating into distinct cell types. Gurniak et al. (2005) showed that the cofilin-mutant mouse embryos fail to form the neural tube and exhibit substantial aberration migration of neural crest cells [66]. Cofilin mutation results in malformation of actin structure and loss of polarity of the neural crest cells, which severely affects neuronal development in mice [66]. Cofilin is indispensable for actin depolymerization and actomyosin organization in the neural epithelium, which are critical for neural tube formation [67,68]. A lack of secretory pathway calcium ATPase (SPCA1) in mouse embryos shares similar neural tube deformation with cofilin mutants [69]. Interestingly, SPCA1 was found to direct cofilin colocalization with apical actin filaments in the neuroepithelium. Thus, cofilin appears as an essential protein for proper neural tube closure during embryonic development.

### 3.2. Neurite Formation

The formation of neurites, the immature projections arising from the neuronal cell body, is a unique and significant step in neurogenesis. The brain’s development and function largely depend on neurite formation, which requires many growth signals, receptor stimuli, and a complex interplay among intracellular and extracellular signals. Actin can function as a microtubule entry barrier in dendritic spines and guide microtubules growing into filopodia [70]. Penetration of microtubules is determined by an adequate balance between forward polymerization and backward transport by the retrograde flow of lamellipodium actin [71]. Three steps are involved in axon elongation: protrusion, engorgement, and consolidation. In protrusion, F-actin’s polymerization triggers elongation of lamellipodia and filopodia, whereas F-actin depolymerization guides polymerized microtubules to elongate into the peripheral domain. In the consolidation step, the transition of microtubules from polymerization to stabilization enables the formation of the neurite shaft. Repeated cycles of these three steps lead to axon elongation. Again, the growth cone’s lamellipodial extension and filopodial retraction are necessary for all three axon elongation stages [71]. Cofilin is highly concentrated in dendritic spines and growth cones of neurons [72]. It controls the number and length of filopodia in response to brain-derived neurotrophic factor (BDNF) [73]. At the rear of actin meshwork in the growth cone, cofilin promotes actin monomers’ recycling to the leading edge for assembly. Cofilin thereby enhances membrane protrusion by altering interactions of F-actin with microtubules [74]. Cofilin also facilitates bundling and penetration of microtubules into the growth cone and restricts microtubule entry into dendritic spines.

Furthermore, several proteins such as neuronal Nogo-A, semaphorin 3A, and BDNF have been found to regulate the growth cone through cofilin [74,75]. Overexpression of cofilin or its phosphorylation-resistant mutant cofilin S3A (active cofilin mutant) can stimulate more growth cone-like waves, which produce significantly longer axons. In contrast, the inactivation of cofilin by LIMK1 overexpression disrupts the fan-like structure of the growth cone, perturbing axon elongation and growth cone motility [76,77]. Such defects can be recovered by overexpressing S3A or slingshot homolog (SSH), a protein phosphatase [51]. Enhancement of actin filament turnover in vivo is critically regulated by cofilin during neurite formation. Cofilin knockout mice exhibit severe abnormalities in multiple brain regions resulting from a profound retrograde flow reduction [76].

### 3.3. Synaptic Plasticity

Long-term potentiation (LTP) and long-term depression (LTD) render durable synaptic plasticity essential for learning and memory. For long-term plasticity, actin-dependent cytoskeletal changes are pivotal in mediating qualitative and quantitative alterations of dendritic spines and synapses (reviewed in [78]).

LTP induction in dendritic spines accompanies spine enlargement and translocation of cofilin to spines [79]. In the LTP, dendritic spine upregulation depends on cofilin phosphorylation, while spine downregulation in the LTD relies on Ca^2+^-dependent calcineurin-induced cofilin dephosphorylation [80]. Cofilin is co-localized with CAP2 dimers in the postsynaptic spine, which are required for actin turnover regulation [60]. Functional loss of cofilin causes aberrant spine enlargement, while overaction of cofilin increases actin depolymerization, leading to spine shrinkage or immature spine formation [81,82,83]. Synaptic relocation of AMPA receptors (AMPARs) is crucial for both LTP and LTD [84]. Cofilin has been found to mediate actin dynamics in postsynaptic trafficking of AMPARs following LTP induction [81]. Moreover, the mobility of AMPARs requires cofilin activity during memory extinction, where phosphorylation of cofilin causes impairment in memory extinction [85]. Therefore, optimum cofilin activity is essential to mediate structural and functional changes in synaptic plasticity.

### 3.4. Axon Regeneration

**Figure 3:**
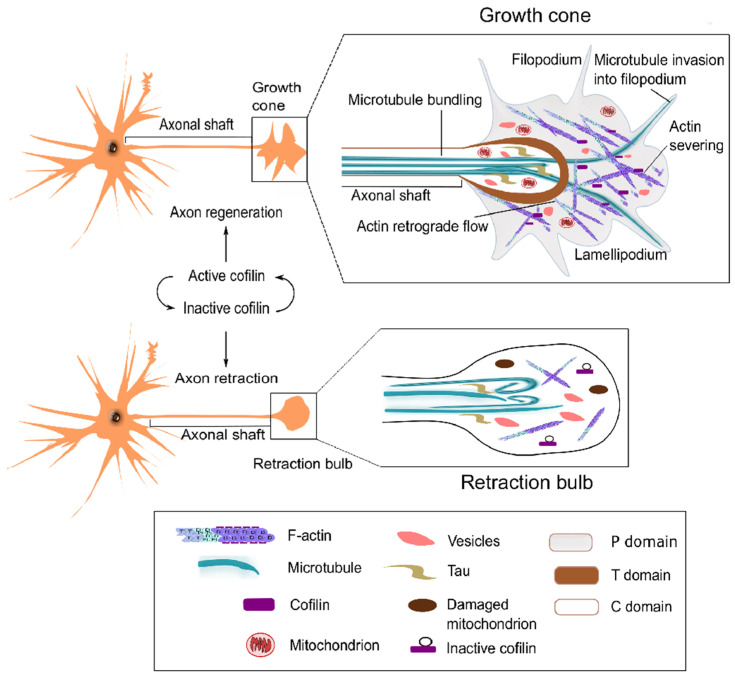
Roles of cofilin in growth cone motility for axonal development. Active cofilin enhances growth cone formation by promoting actin turnover during development and regeneration. Cofilin inactivation suppresses actin turnover, which hinders growth cone development and may cause axonal retraction.

Axonal distortion following external or internal injury or inflammation in the CNS ensues neurodegeneration. Ironically, a failed regeneration of injured axons in the adult CNS is contrasted with the vigorous axonal growth during embryonic development. Regeneration failure is associated with extracellular inhibitory factors and downregulation of neuron-intrinsic regenerative programs (reviewed in [86,87]). Unlike the developing neurons, injured CNS neurons do not display growth cones following axon injury [88]; instead, dystrophic bulbs called ‘retraction bulbs’ are generated (Figure 3).

Stern et al. (2013) studied regeneration events in a mouse nerve injury model where facial axotomy induced nuclear localization of cofilin [89]. Interestingly, activating injured neurons with serum response factor (SRF) enhanced neurite formation and growth cone structures with concomitant depletion of cofilin’s nuclear translocation. Citron kinase (citron-K) is an inhibitor of neuronal regeneration [90]. Knockdown of citron-K increased cofilin levels in rat dorsal root ganglion (DRG) cultures treated with CNS myelin extract and fibroblast growth factor-2 (FGF2). It attenuated neurite outgrowth inhibition of DRG neurons [90]. Tedeschi et al. (2019) showed that nerve conditioning lesions in rodents induce rapid actin turnover and growth cone regeneration in both the PNS and CNS [91]. Conditioning enhanced SSH1 activity and reduced p-cofilin levels, which accompanied a significant increase in the number of growth cone protrusions and filopodia. Intriguingly, conditioning-induced axon regeneration was dependent on the actin severing activity, but not on the depolymerization activity of cofilin [91]. In addition, both actin reorganization and axon regeneration were seen to be abolished in cofilin-deficient neurons after spinal cord injury [91]. Therefore, accumulating evidence positions cofilin as a potential molecular target for CNS axon regeneration.

## 4. Cofilin Dysregulation and Neurodegenerative and Psychiatric Disorders

**Figure 4:**
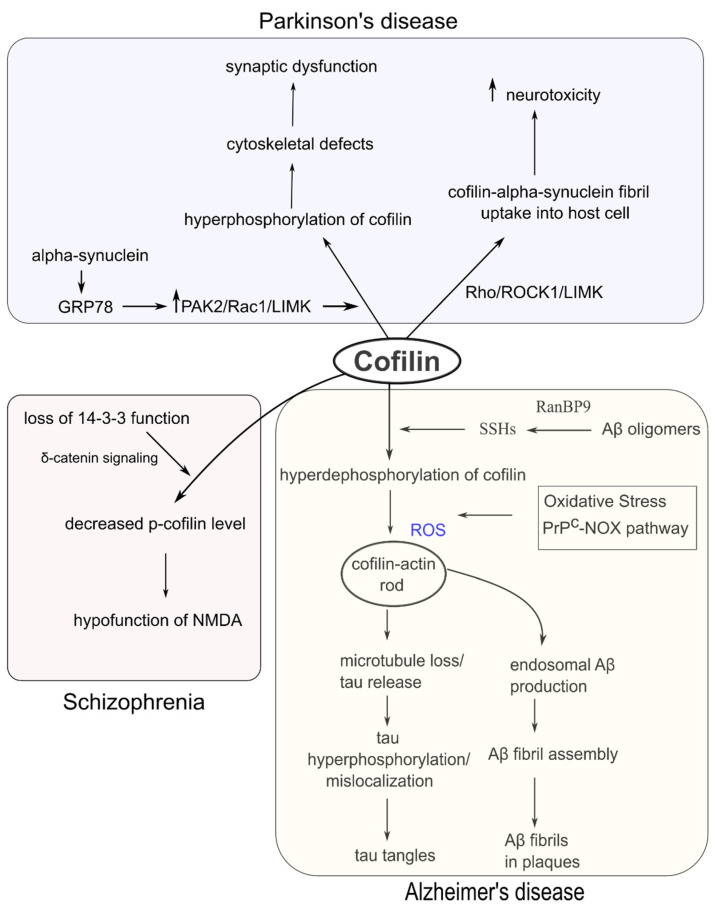
Dysregulation of cofilin in neurodegenerative and psychiatric disorders. Upper (cofilin dysfunction in PD): Alpha-synuclein can induce cofilin hyperphosphorylation leading to synaptic aberration. Alpha-synuclein can also interact with cofilin to form cofilin-alpha-synuclein fibrils and enhance neurotoxicity. Lower left (cofilin dysfunction in schizophrenia): Functional loss of 14-3-3 decreases the p-cofilin level and NMDA function. Lower right (cofilin dysfunction in AD): Aβ oligomers can activate SSHs and induce cofilin hyperdephosphorylation, which enhances cofilin-actin rod formation in the presence of excess ROS. Cofilin-actin rods can further accelerate the formation of tau tangles and Aβ plaques.

Since cofilin is an essential regulator of cytoskeletal and neuronal functions, disruption of its structure and function has profound implications in several neurological disorders (Figure 4). Cofilin dysregulation in rodent models exhibited many neurological symptoms, including cognitive impairment, memory dysfunction, and sleep deprivation [92]. On the other hand, excess phosphorylation of cofilin can induce dendrite reduction and neurodegeneration in Alzheimer’s disease (AD) and schizophrenia [93].

### 4.1. Alzheimer’s Disease (AD)

AD is a chronic neurodegenerative disease that starts slowly and worsens gradually over time. The most common symptoms are associated with signs of dementia and behavioral disorders. Both familial and sporadic AD types can be induced by diverse factors such as aging, oxidative stress, neuroinflammation, and synaptic disruption [94,95]. The two most common pathophysiologic hallmarks of AD are (1) deposition of extracellular beta-amyloid (Aβ) (in senile plaques) and (2) phospho-tau containing intracellular neurofibrillary tangles “tau tangles” (paired helical filaments) (reviewed in [96,97]).

An appearance of amyloid-beta (Aβ)- and tau-dependent spine loss is a pathologic feature that directly correlates with cognitive declines in AD [98]. Cofilin was found to aggregate with Aβ oligomers in human AD brain tissues and mouse AD models [11,99]. Aβ interacts with many synaptic proteins such as NMDA receptors, PrP^C^ (prion protein), ephrin type B-2 receptor (EphB2), metabotropic glutamate receptor 5 (mGluR5), and β-integrin [100]. Woo et al. (2015) showed, using both cultured neurons and in vivo mouse models, that the ran-binding protein 9 (RanBP9) can mediate the accumulation of cofilin-actin rods [101]. RanBP9 enhanced the PrP^C^-dependent Aβ-β1-integrin signal and cofilin dephosphorylation by SSHs. The RanBP9-SSH1-cofilin axis promoted cofilin translocation in the mitochondria and induced a cofilin-actin pathology leading to synaptic and mitochondrial dysfunction [101,102]. Decreased cofilin expression by downregulation of RanBP9 resulted in protection from memory and learning defects in a mouse model of contextual fear conditioning, signifying roles of cofilin activity levels in hippocampal learning and memory [101].

Cofilin activation in cultured neurons induced cofilin-rod formation, transforming phosphorylated microtubule-associated protein tau (MAPT) into cytoskeletal inclusions [103]. In Tau-P301S mice, active cofilin was found to promote tauopathy by specific inhibition of the tau-microtubule interaction through direct competition with tau [104,105]. Moreover, synaptic dysfunction in tau-P301S hippocampal neurons was rescued through genetic ablation of cofilin [104]. In the tau-P301S tauopathy mice, chronic administration of LM11A-31, a small-molecule ligand for p75 neurotrophin receptor (p75NTR), prevented activation of c-Jun N-terminal kinase (JNK) pathway and normalized cofilin phosphorylation at Ser-3 [106]. Notably, LM11A-31 lessened the dendritic spine degeneration and improved hippocampal behaviors, suggesting cofilin association in tauopathies [106].

Decreased CAP2 and increased cofilin levels were reported in hippocampal postsynaptic fractions in an AD mouse model [107]. Small hairpin RNA (shRNA)-mediated down-regulation of CAP2 altered dendritic and spine morphologies in cultured neurons. Additionally, induction of LTP in rat hippocampal neurons augmented CAP2 dimerization and CAP2-cofilin association. This study also found a decreased hippocampal CAP2 protein level and a reduction in the ratio of CAP2/cofilin in AD patients compared to healthy controls, implicating CAPs in AD pathophysiology [107].

### 4.2. Schizophrenia

Schizophrenia is a complex neuropsychiatric disorder with diverse symptoms, including hallucination, delusions, restricted emotion, and other cognitive impairments affecting memory, attention, and executive functions (review [108,109]). Synaptic dysfunctions such as altered synaptic plasticity and synapse formation have been reported in schizophrenia [110,111]. Schizophrenia has been genetically associated with the opioid binding protein/cell adhesion molecule (OPCML), which is abundantly expressed in CNS, especially in the hippocampus and cerebral cortex [112]. OPCML polymorphisms showed association with risks to schizophrenia and some other psychiatric disorders [113]. *Opcml*-deficient (*Opcml*^−/−^) mice also displayed abnormal sensorimotor gating and impaired cognitive behaviors similar to schizophrenia [114]. OPCML was found to interact with EphB2 and control spine stability by regulating the ephrin-EphB2-cofilin signaling pathway. Notably, a pharmacologic intervention with aripiprazole administration restored the abnormal behaviors in *Opcml*^−/−^ mice by increasing p-cofilin and facilitating spine maturation [114]. This report suggests that decreased phosphorylation of cofilin might be involved in schizophrenia pathophysiology.

Another study reported that functional inhibition of 14-3-3 protein in neurons in the mouse nervous system lead to behavioral deficits corresponding to the core symptoms of schizophrenia [115]. A low level of phosphorylated cofilin and NMDA hypofunction were observed in the mutant mice, suggesting that disruption of the 14-3-3 protein function might cause schizophrenia symptoms through an aberration of actin dynamics. The authors suggested that the 14-3-3 protein indirectly regulates p-cofilin level through the δ-catenin signaling pathway rather than altering p-cofilin through a direct protein-protein interaction [115].

### 4.3. Ischemic and Hemorrhagic Stroke

Immediately after ischemic stroke, neuronal cell death occurs rapidly due to initial loss of blood flow, increased oxidative stress by an overload of cytosolic Ca^2+^, increased excitotoxicity, lack of oxygen, and glucose. Overload of Ca^2+^ activates NADPH-oxidase (NOX) through PKC and nitric oxide (NO), leading to a high level of glutamate accumulation in extracellular space (reviewed in [116,117]). Glutamate causes excitotoxicity at higher doses by overstimulating NMDA, AMPA receptors, Ca^2+^ overload, and mitochondrial dysfunction [118]. Several studies reported that both non-NMDA and NMDA receptors can stimulate glutamate-induced cofilin dephosphorylation and rod formation [119,120,121]. During excitotoxic neuronal death, NMDARs stimulation promotes cofilin dephosphorylation by the Ca^2+^-SSH1-cofilin pathway in cortical neurons where cofilin physiologically remains phosphorylated. NMDA-induced cofilin dephosphorylation enhances cofilin translocation in mitochondria and decreases p-cofilin level in the cytosol. Cofilin contributes to the translocation of Bax into mitochondria. [122], promoting mitochondrial membrane depolarization and releasing apoptotic factors like cytochrome C. Acute knockdown of cofilin or SSH1 exhibited a marked neuroprotective action on NMDA-mediated neuronal death [122]. Another recent study demonstrated that rod-induced microtubule-associated protein-2 (MAP2) degradation and cofilin-mediated apoptosis are reduced if cofilin is inhibited by LIMK1 overexpression in the infarct cortex after stroke [123].

Moreover, cofilin oxidation may lead to oxidant-induced apoptosis [124]. Ischemic and hemorrhagic stroke-induced oxidative stress might be a consequence of ROS-induced cofilin oxidation, which translocates free cofilin into the mitochondria, thereby initiates cytochrome C release leading to apoptosis [125]. During the acute phase of ischemic injury and the initial phase of secondary injury to intracranial hemorrhage (ICH), inhibition of elevated cofilin activation in the extracellular region either using pharmacological inhibitor or via phosphorylation could diminish excitotoxicity-induced neuronal death (reviewed in [126]).

Using a rat stroke model, Shu et al. (2019) suggested that rod formation disrupts dendritic mitochondrial trafficking during ischemic conditions [120]. Accumulation of rods induced impaired synaptic structure and blockade of dendritic trafficking in the brain, whereas elevation of p-cofilin level successfully inhibited rod formation and rescued synaptic structure [120].

Pharmacological induction of the 70 kDa heat shock protein (HSP70) has been suggested as a potential therapeutic intervention for stroke [127]. The HSP70-deficient mice exhibited an increased cofilin-actin rod and a larger lesion size in the ischemic border zone. In contrast, HSP70 overexpression in transgenic mice reduced rod formation and improved neurological symptoms following stroke [128]. These reports indicate that cofilin is necessary during the recovery phase of stroke, and perturbed cofilin activity during this period might negatively affect the regenerative process. Therefore, rod suppression via cofilin modulation could emerge as an efficacious treatment approach for ischemic stroke.

### 4.4. Parkinson’s Disease (PD)

PD is another age-related neurodegenerative disorder characterized by the dopaminergic neuron loss in the substantia nigra and by the presence of Lewy neurites (LNs) and Lewy bodies (LBs) in cortical and subcortical neurons [129]. A significant component of LBs and LNs is the misfolded alpha-synuclein (α-synuclein), a conventional protein widely distributed in CNS, mainly in the presynaptic nerve terminals [130]. It has been suggested that α-synuclein exerts its pathologic function in a cell-autonomous manner in the neuronal cytoplasm and may amplify and propagate PD-related pathology [131].

Extracellular α-synucleins upregulate surface-exposed glucose-related protein of 78kDa (GRP78, an endoplasmic reticulum chaperone) that becomes clustered into microdomains of the neuronal plasma membrane [132]. Interaction of α-synucleins and GRP78 activates a signaling cascade, which could phosphorylate/inactivate cofilin and promote actin stabilization and stress fibers formation. Downregulation of GRP78 abolishes α-synuclein-driven cofilin phosphorylation and actin stabilization. Alpha-synuclein has also been correlated with PAK2 and Rac1 phosphorylation, which mediates cofilin phosphorylation via LIMK, implicating the Rac1/PAK2/LIMK/cofilin pathway in α-synuclein-induced actin-alteration [132]. Interestingly, α-synuclein-induced cytoskeletal-associated defects in axonal development could be prevented by cofilin activation in cultured hippocampal neurons [21].

The pathological α-synuclein-fibrils are transmitted from the fibril-generating cells to recipient cells and spread throughout the brain. Cofilin was found to facilitate the α-synuclein-fibril uptake into recipient cells and the expansion of α-synuclein pathology [133]. The combined fibrils consisting of α-synuclein and cofilin are more compact and more potent than aggregated α-synuclein-fibril. Though the underlying mechanism of cofilin-α-synuclein-fibril interaction is unknown, it is deducible that in the presence of α-synuclein-fibrils, cofilin may alter the membrane barrier by depolymerizing actin and form vesicles to promote endocytosis [133]. Upregulation of cofilin by silencing ROCK1 and Rho was found to increase α-synuclein-fibril entry, whereas cofilin downregulation decreased the α-synuclein-fibril entry into the cell [134]. The authors suggested that the Rho-ROCK1-LIMK-cofilin signaling pathway triggers α-synuclein-fibril uptake into the host cells, and the pathogenic impact of α-synuclein in the actin cytoskeleton proceeds through cofilin [134].

Mutations of the parkin (*PARK2*) gene have been linked to autosomal recessive juvenile parkinsonism and early-onset parkinsonism [135,136]. Lim et al. provided evidence on functional interaction between parkin and LIMK1 in human dopaminergic neuroblastoma-derived BE(2)-M17 cell line, where parkin overexpression enhances LIMK1-ubiquitination and reduces the level of LIMK1-induced cofilin phosphorylation [137]. Further research would be necessary to substantiate the roles of cofilin in the pathophysiology of early-onset PD.

## 5. Conclusions

The findings generated from a plethora of studies implicate that proper balance in cofilin activity is a prerequisite for actin turnover and CNS functions. Among various regulatory pathways already discovered for cofilin, phosphoregulation through SSHs and LIMKs is the most critical mechanism, where the Ser-3 residue of cofilin is the specific target. Roles of cofilin Cys-39 and Cys-147 residues are also becoming apparent in the context of ROS-induced rod formation in many neurodegenerative disorders. Newer studies report different molecular pathways of cofilin through which cofilin dysregulation and translocation in subcellular regions might be associated with various CNS disorders.

Recent advances in experimental techniques will significantly facilitate the understanding of the signaling pathways of cofilin function in development and disease conditions. Drugs or peptides targeting the critical amino acid residues of cofilin might be a new potential therapeutic strategy for neurodegenerative disorders.

## Figures and Tables

**Table 1:** Short overview of the functional modulators of cofilin.

Enzymes	Gene Name	Isoforms/Alternative Names	Roles in Cofilin Regulation Pathway	References
Slingshot phosphatase	*SSH*	SSH1 SSH2 SSH3	Dephosphorylate cofilin; dephosphorylate coronins	[31,34]
Coronins	*CORO*	Coronin 1A Coronin 1B Coronin 2A	Induce cofilin activation via SSH1 dephosphorylation, Interact with 14-3-3zeta protein	[35,36]
14-3-3zeta protein	*YWHAZ*	Protein kinase C inhibitor protein 1 (KCIP-1)	Downregulates cofilin activity via SSH1 deactivation and LIMK activation	[37,38]
Protein kinase D enzymes (PKDs)	*PRKD*	PKD1 PKD2 PKD3	Decrease cofilin dephosphorylation by promote 14-3-3zeta protein binding with SSH1 and by inducing LIMK1 activation	[39]
Chronophin (CIN)	*PDXP*	Pyridoxal phosphate phosphatase	Interacts and inhibits Hsp90-mediated LIMK activation, hence induce cofilin phosphorylation	[40,41]
LIM kinases	*LIMK*	LIMK1 LIMK2	Phosphorylate and inactivate cofilin	[42,43]
TES kinases	*TESK*	TESK1 TESK2	Phosphorylate and inactivate cofilin	[44,45]
Reelin	*RELN*	Isoform 1 Isoform 2 Isoform 3	Increase cofilin phosphorylation by inducing LIMK1 activation	[46,47]
SH3 protein interacting with Nck, 90 kDa (SPIN90)	*KCKIPSD*	54 kDa vimentin-interacting protein (VIP54)	Inhibits cofilin activity by binding	[48]
Cortactin	*CTTN*	Amplaxin; oncogene EMS1	Downregulates cofilin activity by binding	[49]
Adenylyl cyclase-associated proteins (CAPs)	*CAP*	CAP1 CAP2	Enhance cofilin activity by synergizing cofilin activity	[50]

## Data Availability

Not applicable.
